# Nanoparticle-based biosensor integrated with multiple cross-displacement amplification for visual and rapid identification of hepatitis B virus and hepatitis C virus

**DOI:** 10.1128/spectrum.01738-24

**Published:** 2025-04-15

**Authors:** Hang Zhang, Yuanfang Shi, Zengguang Wu, Qi Zhao, Yu Wang, Xinggui Yang, Yan Tan, Yi Wang, Zhenghua Xiao, Xu Chen

**Affiliations:** 1 Clinical Laboratory, the Second Affiliated Hospital, Guizhou University of Traditional Chinese Medicine 643558, Guiyang, Guizhou, China; 2 The Second Clinical Medical College, Guizhou University of Traditional Chinese Medicine 326770 , Guiyang, Guizhou, China; 3 Department of Scientific Research, the Second Affiliated Hospital, Guizhou University of Traditional Chinese Medicine 643558, Guiyang, Guizhou, China; 4 Department of Gastroenterology, the Second Affiliated Hospital, Guizhou University of Traditional Chinese Medicine 643558, Guiyang, Guizhou, China; 5 Department of Clinical Laboratory Centre, The First People's Hospital of Guiyang 592766, Guiyang, Guizhou, China; 6 Experimental Center, Guizhou Provincial Centre for Disease Control and Prevention, Guiyang, Guizhou, China; 7 Clinical Laboratory, Guizhou Provincial Center for Clinical Laboratory, Guiyang, Guizhou, China; 8 Experimental Research Center, Capital Institute of Pediatrics 36776, Beijing, Beijing, China; Emory University School of Medicine, Atlanta, Georgia, USA

**Keywords:** hepatitis B virus, hepatitis C virus, biosensor, lateral flow platform, multiple cross displacement amplification

## Abstract

Infection with hepatitis B virus (HBV) and hepatitis C virus (HCV) is a major contributor to liver-related morbidity and mortality worldwide. An accurate and rapid point-of-care (POC) diagnostic approach is the gateway for effective treatment and control of these infections. Here, for the first time, we integrated isothermal multiple cross-displacement amplification (MCDA) with a gold nanoparticle-based lateral flow biosensor (AuNPs-LFB) to successfully develop a novel HBV&HCV-MCDA-AuNPs-LFB assay for simultaneous accurate, sensitive, rapid, inexpensive, and visual identification of HBV and HCV agents. The two unique sets of MCDA degenerate primers were successfully designed targeting the *S* and 5′ untranslated region (5′-UTR) genes from the major HBV genotypes (B, C, D, B/C recombinant, and C/D recombinant) and HCV subtypes in China (1b, 2a, 3a, 3b, and 6a), respectively. The optimal conditions for the MCDA reaction were confirmed to be 64°C for 35 min. The MCDA products were decoded visually using the AuNPs-LFB platform, which was devised for analyzing three targets, including HBV-MCDA, HCV-MCDA amplicons, and a chromatography control. The whole detection procedure, including rapid nucleic acid extraction (~10 min), MCDA reaction (35 min), and AuNPs‐LFB interpretation (~2 min), can be completed within 50 min. The HBV&HCV-MCDA-AuNPs-LFB assay can detect the target genes (HBV-*S* and HCV-5′-UTR) with as low as 10 copies of gene-containing plasmid template per test and does not cross-react with other pathogens. Therefore, our preliminary results indicated that the HBV&HCV-MCDA-AuNPs-LFB assay developed in this study can potentially serve as a useful POC diagnostic tool for the identification of HBV and HCV infections.

**IMPORTANCE:**

Hepatitis B virus (HBV) and hepatitis C virus (HCV) infections have been regarded by the World Health Organization as major threats to human health, especially in low- and middle-income regions. Underdiagnosis of HBV/HCV is a particular challenge for achieving the World Health Organization’s goal of eliminating HBV and HCV infections by 2030. Here, for the first time, we integrated isothermal multiple cross-displacement amplification (MCDA) with a gold nanoparticle-based lateral flow biosensor (AuNPs-LFB) to successfully develop a novel HBV&HCV-MCDA-AuNPs-LFB assay for simultaneous accurate, sensitive, rapid, inexpensive, and visual identification and differentiation of HBV and HCV agents.

## INTRODUCTION

Hepatitis B virus (HBV) and hepatitis C virus (HCV) infections have been regarded by the World Health Organization (WHO) as major threats to human health, especially in low- and middle-income regions (1, 2). It was estimated that in 2019, 295.9 million people were living with chronic HBV infection, and 57.8 million people were living with chronic HCV infection worldwide. Both viruses account for nearly one-fourth of all deaths from infectious diseases worldwide (3, 4). In 2016, the WHO launched the ambitious goal of eliminating viral hepatitis as a public health threat by 2030 and stressed the importance of diagnostic tests and therapy (5). The goal is for 90% of people living with HBV and HCV infections to know their status, and 80% of patients to receive antiviral treatment and be cured (6). However, a WHO report emphasized that less than 1% of chronic HBV-/HCV-infected individuals know about their illness in low- and middle-income settings (1). Underdiagnosis of HBV/HCV is a particular challenge for achieving the WHO’s goal of eliminating HBV and HCV infections by 2030.

Currently, laboratory-based immunoassays and nucleic acid amplification tests (NAATs) are the predominant approaches for HBV/HCV detection in clinical practice (7). Immunoassays are based on a serological response targeting HBV/HCV antibodies or antigens (8). However, these assays may not be suitable for diagnosing acute HBV/HCV infection because they require a long window period (1–6 months)(9). Moreover, patients with immunosuppression can have false-negative results (10). NAATs such as polymerase chain reaction (PCR) and real-time quantitative PCR have been regarded as the new standard techniques of choice for the early detection of HBV/HCV infections in clinical practice owing to their high sensitivity and specificity (11). Nevertheless, these approaches are often inaccessible and unaffordable in resource-constrained regions because they require significant investments in infrastructure, instruments, and training (12). In addition, this process is time-consuming (approximately 2.5 h). Hence, developing an affordable, sensitive, specific, rapid, equipment-free, and user-friendly point-of-care (POC) diagnostic platform is necessary for meeting the WHO goals of viral hepatitis elimination.

Multiple cross-displacement amplification (MCDA) is a novel nucleic acid isothermal amplification approach based on strand-displacement DNA synthesis and auto-cycling that has been validated as an alternative technique to PCR-based methods and is applicable for the identification of specific nucleic acid targets (13–15). MCDA could serve as a promising POC diagnostic platform because it operates at an isothermal temperature and benefits from its high specificity and sensitivity and its simple, rapid, instrument-free, and practical procedure for “on-site” detection (16, 17). MCDA has been used to detect various pathogens, such as monkeypox virus, severe acute respiratory syndrome coronavirus 2, and *Mycobacterium tuberculosis* (18–20). Gold nanoparticle-based lateral flow biosensor (AuNPs-LFB) represents a simple, quick, and inexpensive diagnostic platform for analyzing various samples at the point of care or in the field owing to their high sensitivity, selectivity, low cost, ability to conjugate with biomolecules, and visual interpretation by the naked eye (21–23). They have been extensively employed in food safety, environmental health, agriculture, and biomedicine (24–26).

In the present study, a novel molecular detection assay in which multiplex MCDA was integrated with AuNPs-LFB was developed successfully for rapid, simple, specific, sensitive, inexpensive, and visual diagnosis of HBV and HCV by targeting their *S* and 5′ untranslated region (5′-UTR) genes, respectively (27, 28). The two genes had no homology with other microbial genomes according to BLAST searches of the GenBank database. The feasibility of our assay was validated using serum samples from suspected HBV/HCV-infected patients.

## RESULTS

### Schematic mechanism of the HBV&HCV-MCDA-AuNPs-LFB assay

**Fig 1:**
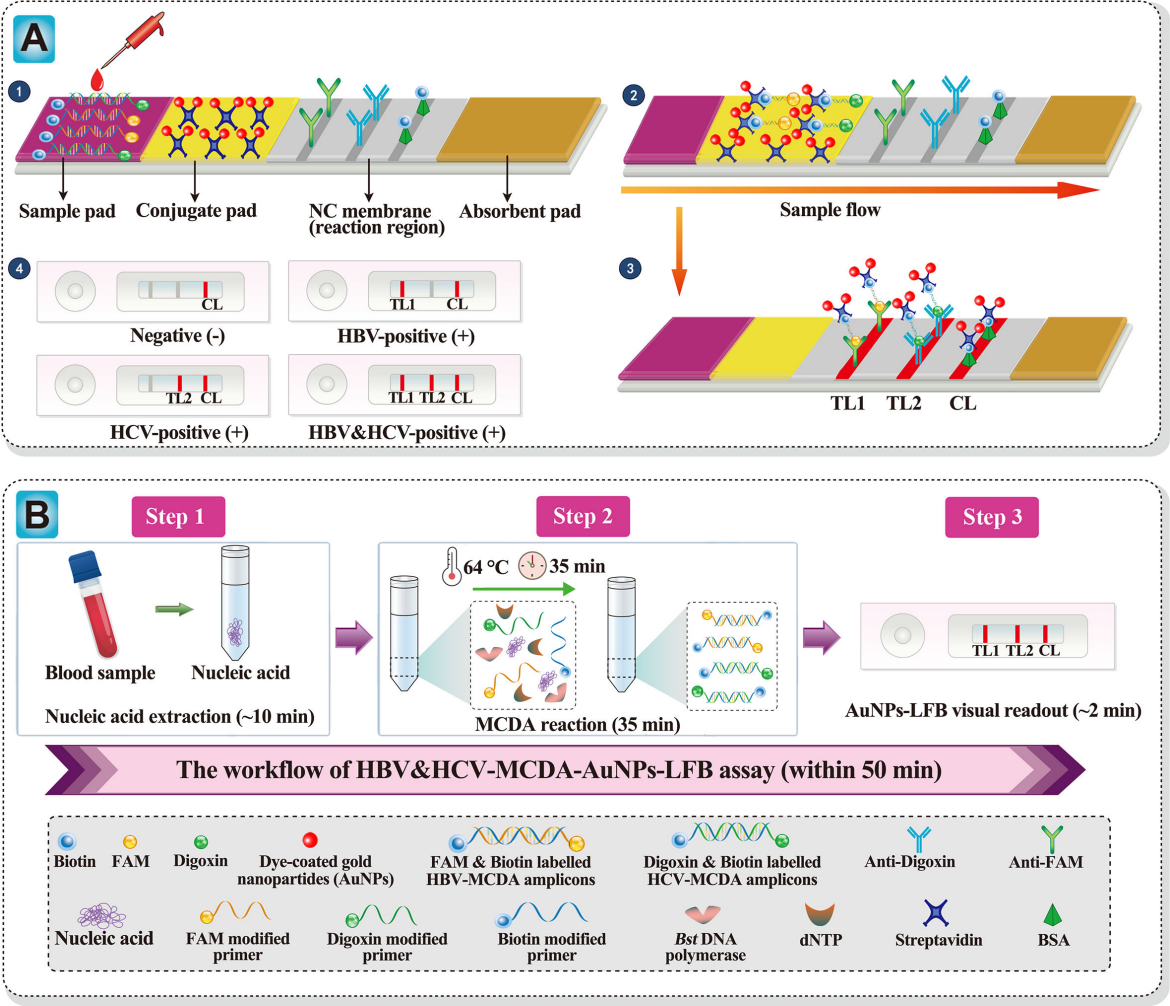
Schematic diagram of HBV&HCV-MCDA-AuNPs-LFB assay′s principle. (**A**) Schematic diagram of AuNPs-LFB principles for the interpretation of HBV&HCV-MCDA products. ❶ Aliquots of 1 µL of HBV&HCV-MCDA products and 100 µL of running buffer (100 mM phosphate buffered saline (PBS), 1% Tween 20, pH 7.4) were simultaneously dripped onto the sample pad region of the biosensor. ❷ The running buffer-containing HBV&HCV-MCDA products moved forward onto the conjugate pad and NC membrane (reaction region) with capillary action. The crimson dye streptavidin-coated gold nanoparticles (streptavidin-AuNPs) are rehydrated and fixed with FAM/biotin-labeled HBV-MCDA products or digoxin/biotin-labeled HCV-MCDA products. ❸ In the reaction region, the FAM/biotin-labeled HBV-MCDA amplicons are arrested by anti-FAM at TL1. Digoxin/biotin-labeled HCV-MCDA amplicons are arrested by anti-digoxin at TL2. Excess dye-coated streptavidin-AuNPs are captured by biotinylated bovine serum albumin (biotin-BSA) at the CL region. ❹ Interpretation of the HBV&HCV-MCDA-AuNPs-LFB assay. HBV-positive results are indicated by CL and TL1 bands on the biosensor. HCV-positive results are indicated by CL and TL2 bands on the biosensor. Both HBV and HCV positive results are indicated by TL1, TL2, and CL bands on the AuNPs-LFB. Negative results are indicated when only the CL band appears on the biosensor. (**B**) Workflow of the HBV&HCV-MCDA-AuNPs-LFB assay. The HBV&HCV-MCDA-AuNPs-LFB assay’s workflow comprises the following closely linked steps: rapid nucleic acid extraction (step 1), MCDA reaction (step 2), and AuNPs-LFB visual readout (step 3). The whole diagnostic procedure can be completed within 50 min.

The details of the AuNPs-LFB analysis of the HBV&HCV-MCDA amplicons are illustrated in Fig. 1A. Aliquots of 1 µL of multiplex-MCDA products and 100 µL of running buffer were simultaneously dripped onto the sample pad region of the biosensor (Fig. 1A, ❶). The running buffer-containing multiplex-MCDA products move along the biosensor via capillary migration. The crimson dye-coated streptavidin gold nanoparticles (SA-AuNPs) were rehydrated by running buffer and then integrated with fluorophore (FAM)/biotin-labeled HBV-MCDA and digoxin/biotin-labeled HCV-MCDA amplicons at the conjugate pad region of the biosensor (Fig. 1A, ❷). At the nitrocellulose (NC) membrane (detection region), the FAM/biotin-labeled HBV-MCDA and digoxin/biotin-labeled HCV-MCDA amplicons were specifically captured by fixed anti-FAM at the first test line (TL1) and immobilized anti-digoxin at the second test line (TL2), respectively. The remaining detector reagents (SA-AuNPs) were captured in the control line (CL) to indicate the effectiveness of the AuNPs-LFB (Fig. 1A, ❸). The interpretation of the multiplex-MCDA results obtained with AuNPs-LFB is presented in Fig. 1A, ❹. An HBV-positive result is obtained when both CL and TL1 turn red. Both CL and TL2 turning red indicate an HCV-positive outcome. CL, TL1, and TL2 simultaneously turning red indicate HBV- and HCV-positive outcomes. A negative result is obtained when only CL turns red.

The workflow of our HBV&HCV-MCDA-AuNPs-LFB assay is shown in Fig. 1B. Briefly, nucleic acids were rapidly extracted from serum samples within 10 min using a DNA/RNA Fast Kit (step 1). Then, multiplex-MCDA reactions were performed at a constant temperature (64°C) for 35 min (step 2). Here, FAM was attached to the HBV *S*-MCDA primer set, and digoxin was added to the HCV 5′-UTR-MCDA primer set. Hence, the HBV *S*-D1* and HBV *S*-C1* primers were labeled at the 5′ end with FAM and biotin, and the HCV 5′-UTR-D1* and HCV 5′-UTR-C1* primers were labeled with digoxin and biotin, respectively. Upon *Bst* 2.0 DNA polymerase activation, the HBV-MCDA amplicons were simultaneously labeled with FAM and biotin, and the HCV-MCDA amplicons were labeled with digoxin and biotin (step 2). The labeled multiplex-MCDA amplicons were analyzed visually with the AuNPs-LFB platform (step 3).

### Validation of the feasibility of the HBV&HCV-MCDA-AuNPs-LFB primer sets

**Fig 2:**
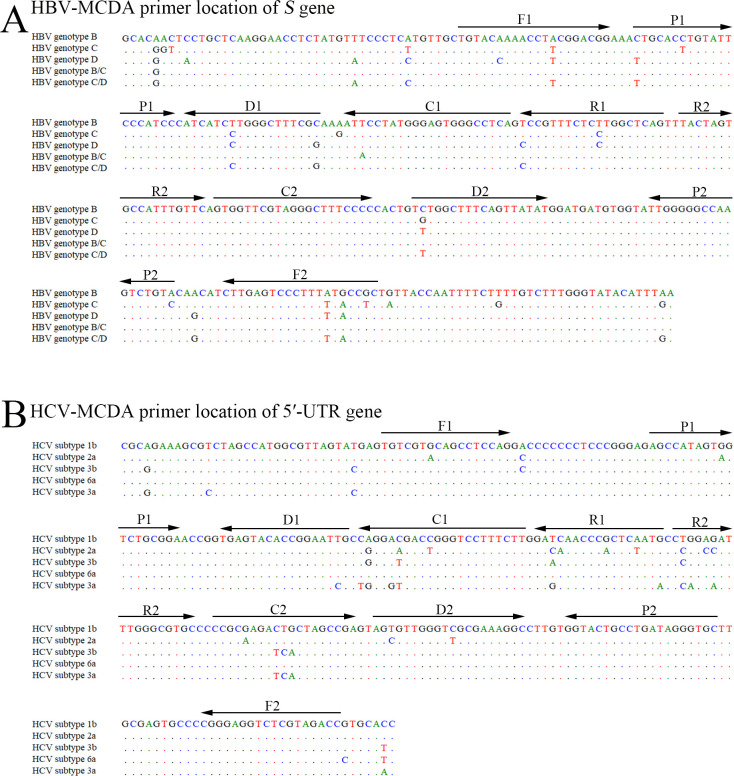
Nucleotide sequences and location of the HBV-*S* and HCV 5′-UTR genes used to design the HBV&HCV-MCDA primers. In the MCDA reaction, the isothermal amplification of specific nucleic acid sequences is achieved by employing a set of 10 primers spanning 10 distinct regions of the target fragment, which are designated as displacement primers F1 and F2, cross primers CP1 (C1 and P1 go together) and CP2 (C2 and P2 go together), and amplification primers (C1, C2, D1, D2, R1, and R2). (**A**) The nucleotide sequences of the *S* gene from five dominant HBV genotypes in China (B, C, D, recombinant B/C, and recombinant C/D) were aligned by DNASTAR software, and the HBV-MCDA primer sequences were marked with arrows. (**B**) The nucleotide sequences of the 5′-UTR gene from five dominant HCV subtypes in China (1b, 2a, 3a, 3b, and 6a) were aligned by DNASTAR software, and the conserved sequences were applied for HCV-MCDA primers. Right arrows and left arrows indicated the sense and complementary sequences which were used in this study, respectively.

**Fig 3:**
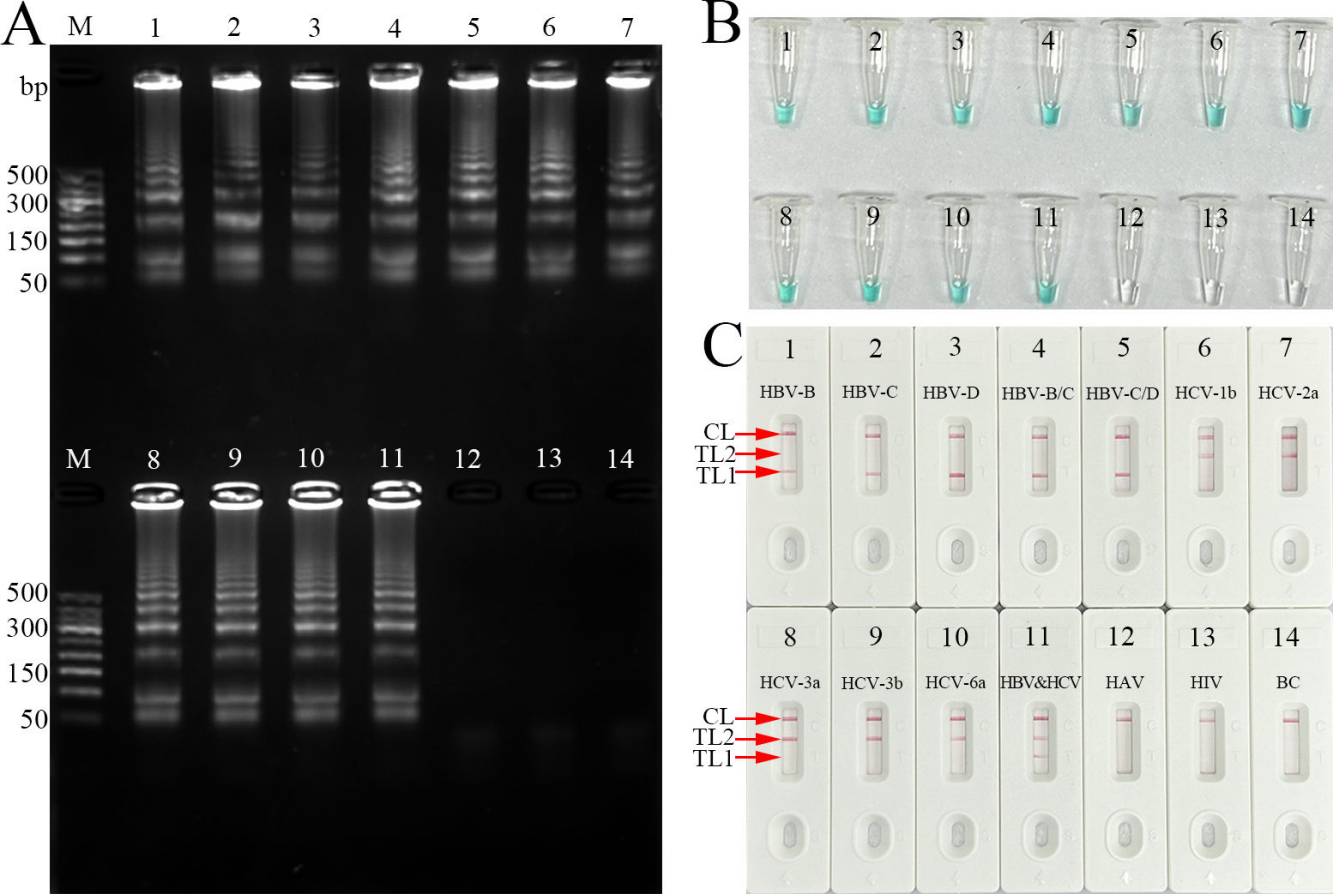
Confirmation and detection of HBV-, HCV-, and HBV&HCV-MCDA products. (**A**) 2% agarose gel electrophoresis, (**B**) visual indicator (MG), (**C**) AuNPs-LFB biosensor. Templates of 1–14 were HBV-B plasmid, HBV-C plasmid, HBV-D plasmid, HBV-B/C plasmid, HBV-C/D plasmid, HCV-1b plasmid, HCV-2a plasmid, HCV-3a plasmid, HCV-3b plasmid, HCV-6a plasmid, HBV&HCV plasmid, HAV, and HIV, respectively. For agarose gel electrophoresis detection, the agarose gel presented ladder-like bands indicating a positive outcome, whereas the lack of bands indicated a negative result. For visual MG analysis, changing the reaction mixture to light green suggested a positive outcome, whereas colorless mixtures indicated a negative result. For AuNPs-LFB identification, both the CL and TL1 simultaneously appeared on the AuNPs-LFB strip, demonstrating an HBV-positive result. Both the CL and TL2 simultaneously turned red on the biosensor, indicating an HCV-positive outcome. For a negative outcome, only CL was present on the AuNPs-LFB strips.

To confirm the feasibility of the HBV- and HCV-MCDA primer sets in our study (Fig. 2A and B; Table 1). Five standard HBV-*S* plasmids (genotypes B, C, D, recombinant B/C, recombinant C/D), five standard HCV-5′-UTR plasmids (subtypes 1b, 2a, 3b, 6a, and 3a), viral RNA from hepatitis A virus (HAV) and human immunodeficiency virus (HIV) were used as templates for the MCDA assay at a fixed temperature of 65°C for 60 min, and distilled water was used as a blank control (BC). Agarose gel electrophoresis, colorimetric indicator (malachite green, MG), and AuNPs‐LFB analysis methods were utilized to interpret the multiplex-MCDA results. The templates from the HBV and/or HCV strand plasmids were amplified and yielded positive results according to each analysis approach (Fig. 3A through C). Importantly, the AuNPs‐LFB method used in the present study can simultaneously detect two targets (HBV and HCV) in a single test (Fig. 3C). No amplification was detected for the negative controls (HAV and HIV) or the blank control distilled water (DW) (Fig. 3). These findings confirmed that the two sets of MCDA primers for HBV and HCV detection were valid for the development of the HBV&HCV-MCDA-AuNPs-LFB assay.

**TABLE 1:** The HBV&HCV-MCDA-AuNPs-LFB degenerate MCDA primers used in this study

Primer name^*a*^	Sequence and modifications^*b*^	Length^*c*^	Gene
HBV-F1	5´-TGTACMAAACCTWCGGACGG-3´	20 nt	*S*
HBV-F2	5´-GMGGYAWAAAGGGACTCAAG-3´	20 nt	
HBV-CP1	5´-TGAGGCCCACTCCCATAGGWATYTGCACYTGTATTCCCATCC-3´	42 mer	
HBV-CP2	5´-GTGGTTCGTAGGGCTTTCCCCKACAGACTTGGCCCCCAA-3´	39 mer	
HBV-C1	5´-TGAGGCCCACTCCCATAGGWAT-3´	22 nt	
HBV-C1*	5´-Biotin-TGAGGCCCACTCCCATAGGWAT-3´	22 nt	
HBV-C2	5´-GTGGTTCGTAGGGCTTTCCCC-3´	21 nt	
HBV-D1	5´-SCGAAAGCCCARGATGAT-3´	18 nt	
HBV-D1*	5´-FAM-SCGAAAGCCCARGATGAT-3´	18 nt	
HBV-D2	5´-TBTGGCTTTCAGTTATAT-3´	18 nt	
HBV-R1	5´-CTGAGCCARGAGAAACGGR-3´	19 nt	
HBV-R2	5´-TACTAGTGCCATTTGTTC-3´	18 nt	
HCV-F1	5´-TGTCGTRCAGCCTCCAG-3´	17 nt	5´UTR
HCV-F2	5´-GGTCTACGAGACCTCCCG-3´	18 nt	
HCV-CP1	5´-AAGAAAGGACCCRGTCDYCCYRAGCCATAGTRGTCTGCGGA-3´	41 mer	
HCV-CP2	5´-CCGCRAGAYYRCTAGCCGAGCACCCTATCAGGCAGTACC-3´	39 mer	
HCV-C1	5´-AAGAAAGGACCCRGTCDYCCYR-3´	22 nt	
HCV-C1*	5´-Biotin-AAGAAAGGACCCRGTCDYCCYR-3´	22 nt	
HCV-C2	5´-CCGCRAGAYYRCTAGCCGA-3´	19 nt	
HCV-D1	5´-CRATTCCGGTGTACTCA-3´	17 nt	
HCV-D1*	5´-Digoxin-CRATTCCGGTGTACTCA-3´	17 nt	
HCV-D2	5´-AGYGTTGGGTYGCGAAAGGC-3´	20 nt	
HCV-R1	5´-YATWGAGYGGGTTKNTC-3´	17 nt	
HCV-R2	5´-CYRGMVATTTGGGCGTGC-3´	18 nt	

^*a*^ HBV-C1*, 5´-labeled with biotin; HBV-D1*, 5´-labeled with FAM; HCV-C1*, 5´-labeled with biotin; HCV-D1*, 5´-labeled with digoxin; when used for the AuNPs-LFB assay.

^*b*^ IUPAC code for mix bases: R, A/G; Y, C/T; M, A/C; K, G/T; S, C/G; W, A/T; H, A/C/T; B, C/G/T; V, A/C/G; D, A/G/T; N, A/C/G/T.

^*c*^ nt, nucleotide; mer, monomeric unit.

### The optimal reaction temperature for the MCDA assay

**Fig 4:**
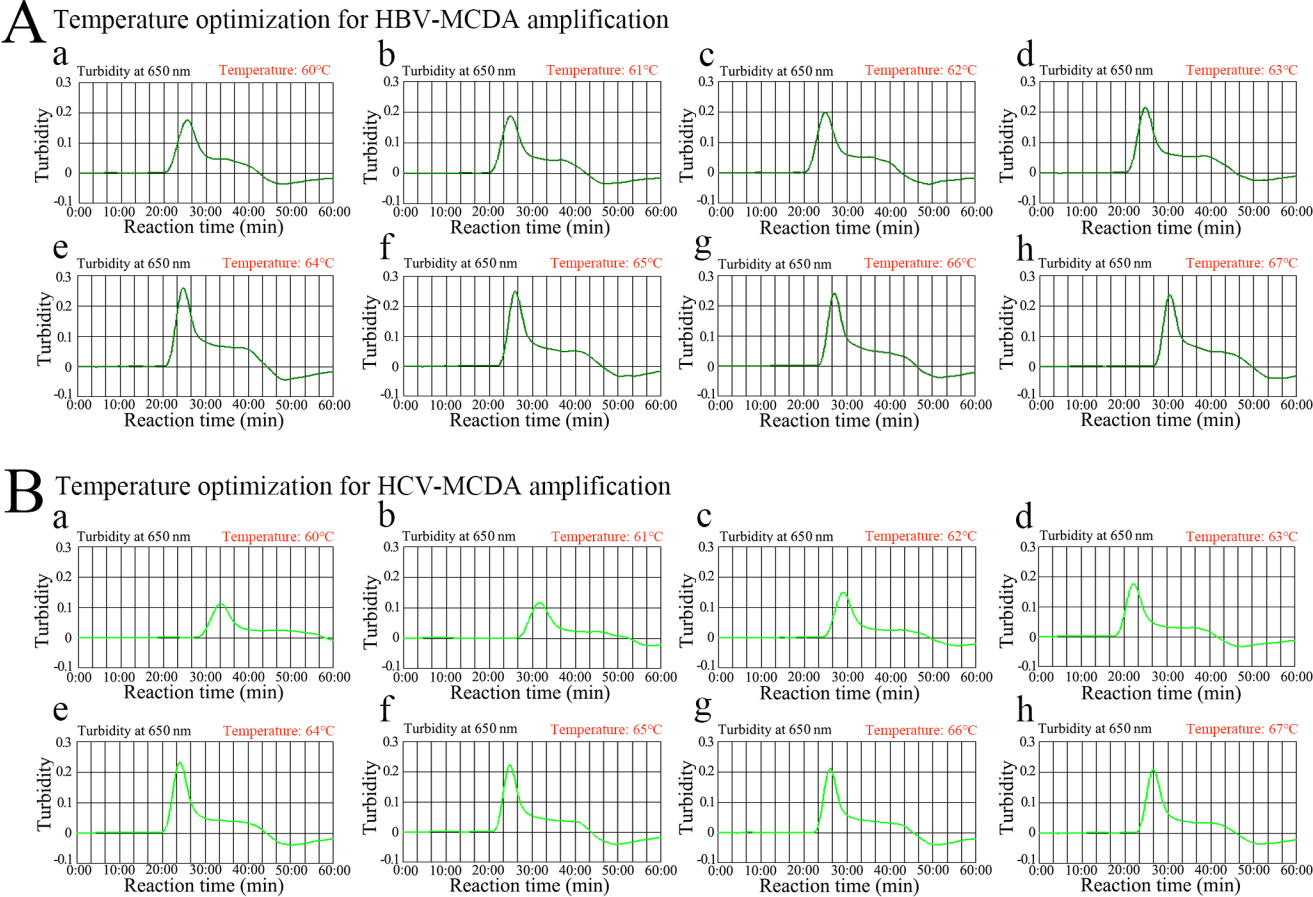
Temperature optimization for HBV- and HCV-MCDA amplification. The MCDA reactions for detection of HBV (**A**) and HCV (**B**) were monitored using real-time turbidity, and their corresponding amplicon curves were shown as graphs. A turbidity >0.1 indicated a positive result. Eight kinetic graphs (a–h) were obtained at different temperatures (60°C–67°C, 1°C increments) with 1 × 10^4^ target gene copies. Graphs e (64°C) to h (67°C) in A showed robust amplification. Graphs from e (64°C) to f (65°C) in B showed robust amplification.

To confirm the optimal temperature for the HBV- and HCV-MCDA assays, the HBV- and HCV-MCDA reactions were regulated at temperatures ranging from 63 to 70°C at 1°C intervals. The standard plasmids (HBV-*S* and HCV-5′-UTR) were used as templates at a concentration of 1.0 × 10^4^ copies per test. The HBV-MCDA and HCV-MCDA reactions were monitored using real-time turbidimetry, and the kinetics graphs corresponding to the different temperatures were plotted (Fig. 4A and B). Robust HBV-MCDA and HCV-MCDA reactions occurred at 64 to 66°C (Fig. 4A) and 64 to 65°C (Fig. 4B), respectively. Hence, 64°C was regarded as the optimal amplification temperature for the HBV&HCV-MCDA-AuNPs-LFB system.

### Analytical sensitivity of the HBV&HCV-MCDA-AuNPs-LFB assay

**Fig 5:**
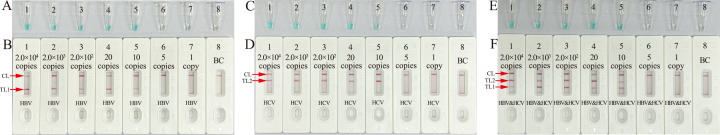
Sensitivity analysis of HBV&HCV-MCDA-AuNPs-LFB assay with serial nucleic acid template dilutions. Serial dilutions of HBV-B and HCV-1b plasmids were used as templates, and distilled water was used as the BC. Results were simultaneously analyzed by visual reagent MG and AuNPs-LFB biosensor. (**A, B**) Sensitivity analysis of HBV-MCDA reaction. Tubes A1–A8 (biosensor B1–B8) represent the HBV-*S* plasmid amounts of 2.0 × 10^4^ copies, 2.0 × 10^3^ copies, 2.0 × 10^2^ copies, 20 copies, 10 copies, 5 copies, 1 copy per reaction, and blank control, respectively. The LoD of HBV-MCDA assay was 10 copies per reaction. (**C, D**) Sensitivity analysis of HCV-MCDA reaction. Tubes C1–C8 (biosensor D1–D8) represent the HCV-5′-UTR plasmid amounts of 2.0 × 10^4^ copies, 2.0 × 10^3^ copies, 2.0 × 10^2^ copies, 20 copies, 10 copies, 5 copies, 1 copy per reaction, and blank control, respectively. The LoD of the HCV-MCDA assay was 10 copies per reaction. (**E, F**) Sensitivity analysis of HBV&HCV-MCDA reaction. Tubes E1–E8 (biosensor F1–F8) represent the HBV&HCV plasmid amounts of 2.0 × 10^4^ copies, 2.0 × 10^3^ copies, 2.0 × 10^2^ copies, 20 copies, 10 copies, 5 copies, 1 copy per reaction, and blank control, respectively. The LoD of HBV&HCV-MCDA assay was 10 copies per reaction. For visual MG analysis, changing the reaction mixture to light green suggested a positive outcome, whereas colorless mixtures indicated a negative result. For AuNPs-LFB identification, both the CL and TL1 simultaneously appeared on the AuNPs-LFB strip, demonstrating an HBV-positive result. Both the CL and TL2 simultaneously turned red on the biosensor, indicating an HCV-positive outcome. For a negative outcome, only CL was present on the AuNPs-LFB strips.

Serial dilutions of the HBV-genotype-B-*S* plasmid and HCV-subtype-1b-5′-UTR plasmid were used as templates to confirm the limit of detection (LoD) of the multiplex-MCDA-AuNPs-LFB assay. The MCDA assay was performed at 64°C for 1 h, and the amplicons were analyzed using AuNPs-LFB and MG reagent. As shown in Fig. 5A through F, the LoD of the MCDA-AuNPs-LFB assay was 10 copies per test for both the HBV-*S* and HCV-5′-UTR gene-containing plasmids (Fig. 5A through D). The sensitivity of multiplex-MCDA-AuNPs-LFB was consistent with that of the single HBV-AuNPs-LFB and HCV-AuNPs-LFB assays (Fig. 5E and F), and the results obtained with the AuNPs-LFB method (Fig. 5B, D, and F) were in accordance with those obtained with the MG reagent (Fig. 5A, C, and E). Notably, the multiplex-MCDA-AuNPs-LFB platform can simultaneously detect the HBV and HCV agents in a single reaction.

### The optimal reaction time for the HBV&HCV-MCDA-AuNPs-LFB assay

**Fig 6:**
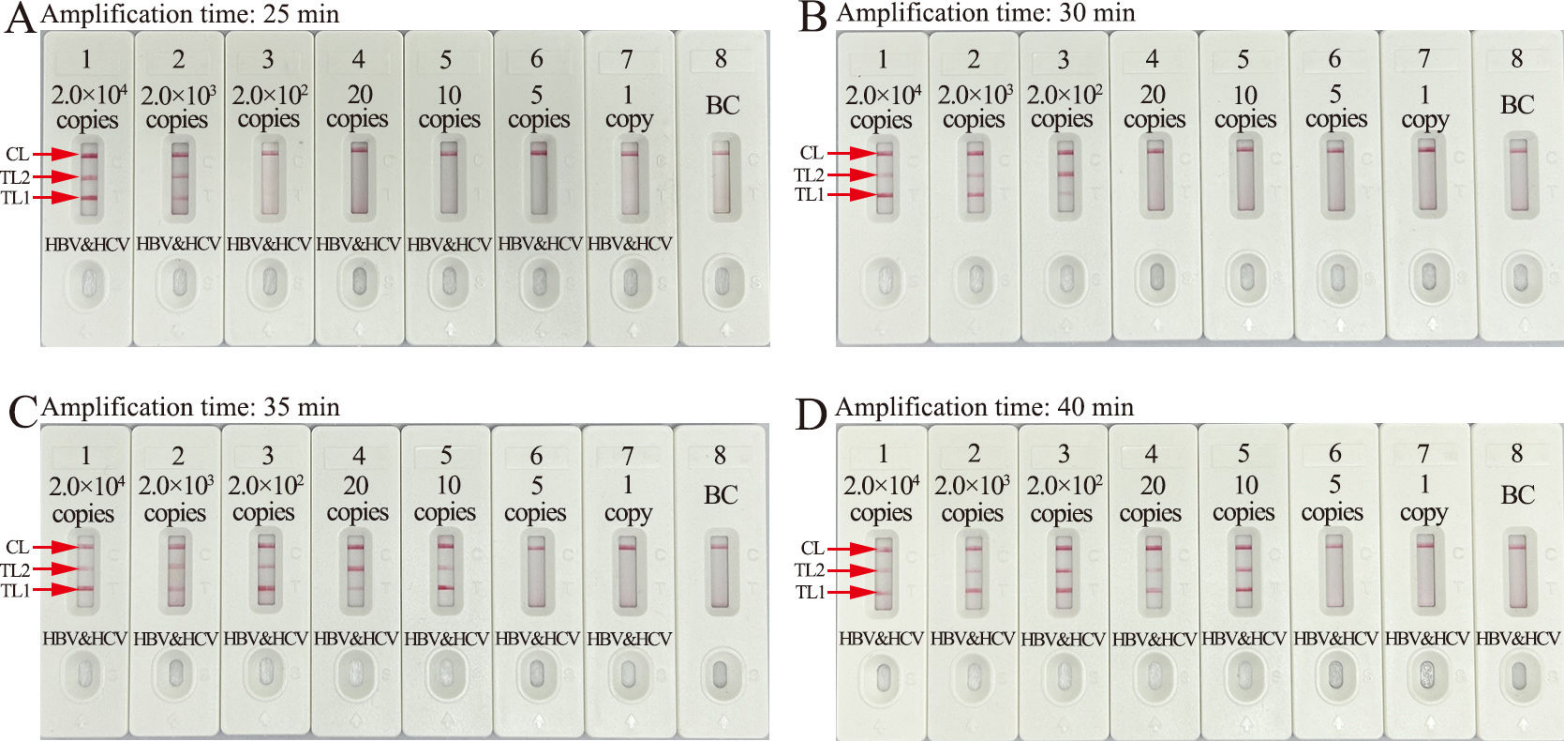
Amplification time optimization for HBV&HCV-MCDA-AuNPs-LFB assay. Different amplification times (A, 25 min; B, 30 min; C, 35 min; D, 40 min) were evaluated at optimal reaction temperature (64℃). Biosensors 1–8 represent HBV-B and HCV-1b nucleic acid template levels of 2.0 × 10^4^ copies, 2.0 × 10^3^ copies, 2.0 × 10^2^ copies, 20 copies, 10 copies, 5 copies, 1 copy, and blank control (distilled water), respectively. HBV&HCV-MCDA amplicons were analyzed using AuNPs-LFB biosensor. The optimal LoD occurred with a 35 min amplification time (**C**).

The optimal reaction time for the amplification stage of the multiplex-MCDA-AuNPs-LFB assay was confirmed with the HBV-genotype-B-*S* plasmid and HCV-subtype-1b-5′-UTR plasmid at the LoD level (10 copies each). All tests were performed at the optimal reaction temperature (64°C), and the results were analyzed using AuNPs-LFB. As shown in Fig. 6, the LoDs of the two plasmid templates could be accurately identified within 35 min of incubation. Hence, the optimal amplification conditions for our HBV&HCV-MCDA-AuNPs-LFB assay were 64°C for 35 min.

To confirm the LoD and reaction time are consistent among different HBV/HCV genotypes. Four other HBV genotypes (C, D, recombinant B/C, recombinant C/D) and four HCV subtypes (2a, 3a, 3b, and 6a) plasmid templates were tested under the optimal reaction conditions (64°C, 35 min), and the results showed that the LoD of each HBV/HCV genotype was 10 copies per test (Fig. S1).

### Specificity of the HBV&HCV-MCDA-AuNPs-LFB assay

**Fig 7:**
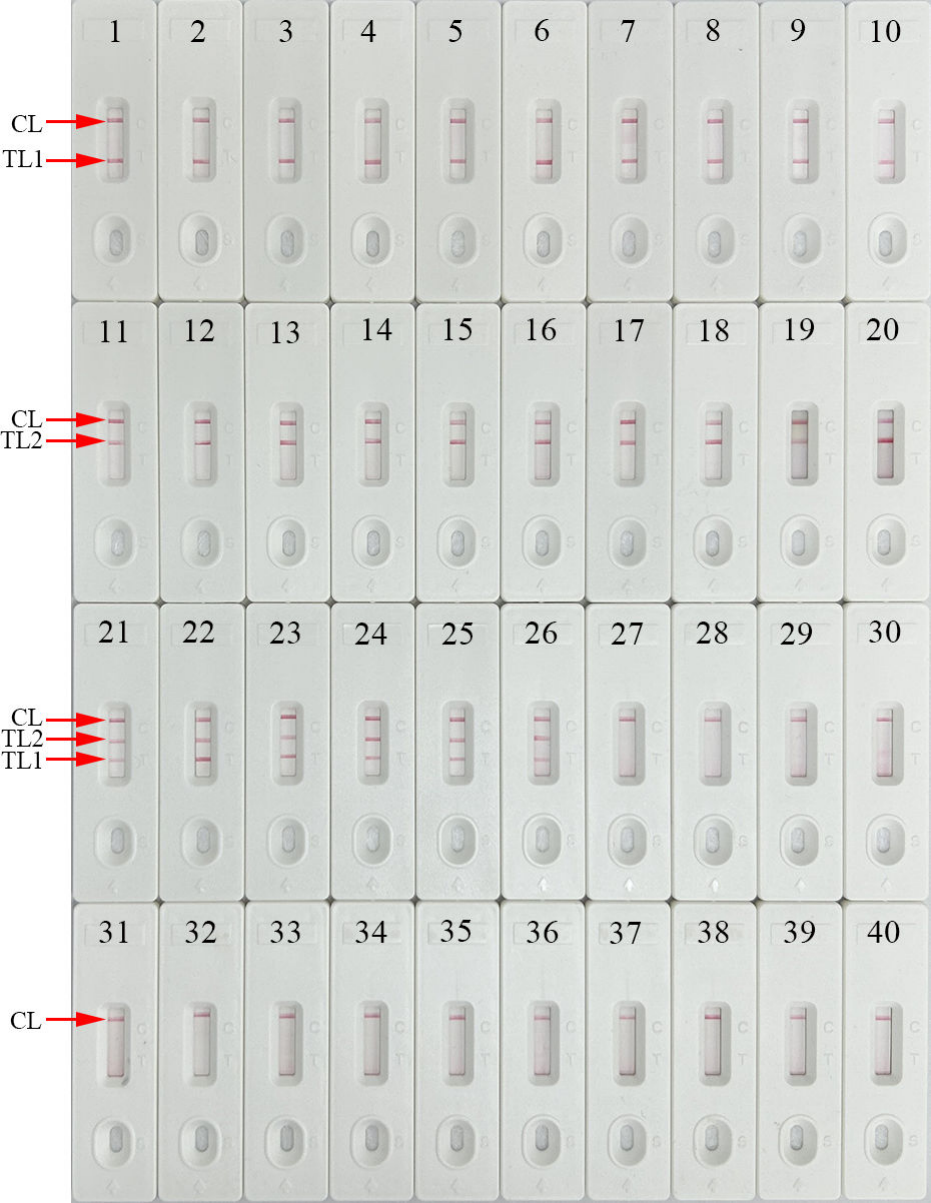
Specificity analysis of HBV&HCV-MCDA-AuNPs-LFB assay with different strains. The MCDA reactions were performed using different nucleic acid templates, and each of the amplification products was analyzed through visual AuNPs-LFB biosensor. Biosensor 1–5, HBV genotype B plasmid, HBV genotype C plasmid, HBV genotype D plasmid, HBV genotype B/C plasmid, HBV genotype C/D plasmid, respectively; biosensor 6–10, HBV strains (clinical samples); biosensor 11–15, HCV subtype 1b plasmid, HCV subtype 2a plasmid, HCV subtype 3b plasmid, HCV subtype 6a plasmid, HCV subtype 3a plasmid, respectively; biosensor 16–20, HCV strains (clinical samples); biosensor 21, HBV&HCV plasmids; biosensor 22–26, HBV&HCV strains (clinical samples); biosensor 27, herpes simplex virus; biosensor 28, parainfluenza virus; biosensor 29, HAV; biosensor 30, coxsackievirus CAV16; biosensor 31, human enterovirus EV71; biosensor 32, influenza A virus; biosensor 33, influenza B virus; biosensor 34, human papilloma virus; biosensor 35, HIV; biosensor 36, *Mycobacterium leprae*; biosensor 37, *Mycobacterium tuberculosis*; biosensor 38, *Haemophilus influenzae*; biosensor 39, *Cryptococcus neoformans*; biosensor 40, blank control (distilled water).

The specificity and selectivity of our assay were validated using HBV-*S* plasmids, HCV-5′-UTR plasmids, positive HBV and/or HCV clinical samples (confirmed by qPCR), 13 other pathogens, and their mixtures (Table 2). The HBV&HCV-MCDA-AuNPs-LFB assay was performed under the optimal reaction conditions (64°C for 35 min), and the results were analyzed using the AuNPs-LFB assay. As shown in Fig. 7 and Table 2, the specificity of our assay was 100% for HBV and HCV detection. Positive HBV and HCV results are indicated by the presence of three red lines (CL, TL1, and TL2) on the biosensor. Positive HBV strains caused the CL and TL1 bands of the biosensor to turn red, and positive HCV strains caused the CL and TL2 bands of the biosensor to turn red. The other pathogens and the blank control had negative results, and no cross-reactions were observed in the current study. These data indicated that our HBV&HCV-MCDA-AuNPs-LFB assay exhibited high specificity.

**TABLE 2:** Microbial strains used in the current study

No.	Strains/Templates	Source of strain^*a*^	No. of strains	HBV&HCV-MCDA-AuNPs-LFB result^*b*^	
				HBV	HCV
1	HBV genotype B *S*-plasmid	Tsingke Biotech (Beijing, China)	1	P	N
2	HBV genotype C *S*-plasmid	Tsingke Biotech (Beijing, China)	1	P	N
3	HBV genotype D *S*-plasmid	Tsingke Biotech (Beijing, China)	1	P	N
4	HBV genotype B/C recombinant *S*-plasmid	Tsingke Biotech (Beijing, China)	1	P	N
5	HBV genotype C/D recombinant *S*-plasmid	Tsingke Biotech (Beijing, China)	1	P	N
6	HBV strains (clinical samples)	2nd GZUTCM	5	P	N
7	HCV subtype 1b 5´UTR-plasmid	Tsingke Biotech (Beijing, China)	1	N	P
8	HCV subtype 2a 5´UTR-plasmid	Tsingke Biotech (Beijing, China)	1	N	P
9	HCV subtype 3b 5´UTR-plasmid	Tsingke Biotech (Beijing, China)	1	N	P
10	HCV subtype 6a 5´UTR-plasmid	Tsingke Biotech (Beijing, China)	1	N	P
11	HCV subtype 3a 5´UTR-plasmid	Tsingke Biotech (Beijing, China)	1	N	P
12	HCV strains (clinical samples)	2nd GZUTCM	5	N	P
13	HBV and HCV plasmids	Tsingke Biotech (Beijing, China)	1	P	P
14	HBV and HCV strains (clinical samples)	2nd GZUTCM	5	P	P
15	Herpes simplex virus	GZCCL	1	N	N
16	Parainfluenza virus	GZCCL	1	N	N
17	Hepatitis A virus (HAV)	2nd GZUTCM	1	N	N
18	Coxsackievirus CAV16	GZCDC	1	N	N
19	Human enterovirus EV71	GZCDC	1	N	N
20	Influenza A virus	GZCDC	1	N	N
21	Influenza B virus	GZCDC	1	N	N
22	Human papilloma virus	GZCCL	1	N	N
23	Human immunodeficiency virus	GZCCL	1	N	N
24	*Mycobacterium leprae*	GZCDC	1	N	N
25	*Mycobacterium tuberculosis*	GZCDC	1	N	N
26	*Haemophilus influenzae*	ATCC49247	1	N	N
27	*Cryptococcus neoformans*	2nd GZUTCM	1	N	N

^*a*^ ATCC, American Type Culture Collection; 2nd GZUTCM, the Second Affiliated Hospital, Guizhou University of Traditional Chinese Medicine; GZCCL, Guizhou Provincial Center for Clinical Laboratory; GZCDC, Guizhou Provincial Center for Disease Control and Prevention.

^*b*^ P, positive; N, negative.

### Feasibility of the HBV&HCV-MCDA-AuNPs-LFB assay in clinical specimens

To verify the suitability of the multiplex-MCDA-AuNPs-LFB assay as a valuable tool for HBV and HCV identification, a total of 107 serum samples were tested simultaneously with our assay and real-time PCR assay, respectively. The results revealed that 48 serum samples (48/107) were HBV positive, 31 samples (31/107) were HCV positive, and 12 samples (12/107) were diagnosed with HBV and HCV coinfections with the real-time PCR approach. All HBV/HCV-positive or -negative samples were confirmed using our assay (Tables 3 and 4; Table S1; Fig. S2). Comparing with the HBV-qPCR technology, the HBV&HCV-MCDA-AuNPs-LFB sensitivity and specificity were 100.00% (95% CI: 94.04% to 100.00%) and 100% (95% CI: 92.45% to 100.00%), respectively (Table 3). Comparing with the HCV-qPCR technology, the HBV&HCV-MCDA-AuNPs-LFB sensitivity and specificity were 100.00% (95% CI: 91.78% to 100.00%) and 100% (95% CI: 94.40% to 100.00%), respectively (Table 4). These data indicated that our assay can be used as a powerful tool for the identification of HBV and HCV infections in clinical settings.

**TABLE 3:** Comparing HBV levels in clinical samples using our HBV&HCV-MCDA-AuNPs-LFB assay and qPCR method

HBV&HCV-MCDA-AuNPs-LFB	HBV-qPCR (reference method)			Sensitivity		Specificity	
	Positive	Negative	Total	Value	95% CI	Value	95% CI
Positive	60	0	60	100	94.04%–100%	100	92.45%–100%
Negative	0	47	47				
Total	60	47	107				

**TABLE 4:** Comparing HCV levels in clinical samples using our HBV&HCV-MCDA-AuNPs-LFB assay and qPCR method

HBV&HCV-MCDA-AuNPs-LFB	HCV-qPCR (reference method)			Sensitivity		Specificity	
	Positive	Negative	Total	Value	95% CI	Value	95% CI
Positive	43	0	43	100	91.78%–100%	100	94.40%–100%
Negative	0	64	64				
Total	43	64	107				

## DISCUSSION

Here, a novel HBV&HCV-MCDA-AuNPs-LFB point-of-care diagnostic platform, which integrated multiple-MCDA isothermal amplification with a visual AuNPs-LFB interpretation platform, was successfully developed for highly accurate, sensitive, rapid, simple, affordable, and visual diagnosis of HBV and HCV infections in a single test. The feasibility of our assay was verified using serum samples from patients with suspected HBV and HCV infections, and the results were compared with those of real-time PCR.

HBV and HCV infections affect several hundred million people and pose a significant burden to the public health system (29, 30). More importantly, the vast majority of HBV or HCV infections have not been diagnosed, especially in low- and middle-income regions (31). Hence, developing an affordable, easy-to-operate, accurate, and sensitive diagnostic platform for HBV/HCV identification is critical for prescribing more effective antiviral treatments and preventing HBV/HCV transmission. Our HBV&HCV-MCDA-AuNPs-LFB assay utilizes a novel multiple-MCDA method for rapidly amplifying target genes within 35 min at a constant temperature (64°C). The MCDA reaction can be performed isothermally using simple devices, such as metal baths, water baths, and heating blocks. The MCDA amplicons were rapidly and visually analyzed using the AuNPs-LFB platform. The crude nucleic acid could meet the need for MCDA isothermal amplification owing to the use of *Bst* DNA polymerase, which has fewer inhibitors than *Taq* DNA polymerase used in traditional PCR systems (27, 32). Hence, our HBV&HCV-MCDA-AuNPs-LFB assay is user-friendly and time-saving, and the whole detection process, including rapid nucleic acid extraction (approximately 10 min), MCDA (35 min), and AuNPs‐LFB interpretation (less than 2 min), can be completed within 50 min.

The HBV- and HCV-specific amplicons were simultaneously generated in the multiplex-MCDA reaction system using only *Bst* 2.0 DNA polymerase with strand-displacement activity and two MCDA degenerate primer sets targeting the HBV-*S* and HCV-5′-UTRs, respectively. The principle of MCDA has been reported previously (15, 18). For validating the feasibility of the HBV&HCV-MCDA-AuNPs-LFB assay, the full-length *S* sequences from five dominant HBV genotypes (B, C, D, recombinant B/C, recombinant C/D) and the HCV 5′-UTR target genes from five dominant subtypes (1b, 2a, 3a, 3b, and 6a) in China were synthetically produced, cloned, and inserted into pUC57 plasmids, respectively. These target gene-containing plasmids were used as positive controls. As the previous studies show, synthetic plasmids containing target sequences can be applied to positive controls for validation of nucleic acid detection technologies (33–35). The condition of our assay at the MCDA reaction stage was optimized to 64°C for 35 min. The specificity of our assay was verified using several HBV genotype strains (B, C, D, B/C recombinant, and C/D recombinant), HCV subtype strains (1b, 2a, 3b, 6a, and 3a), and other pathogens, and the HBV&HCV-MCDA-AuNPs-LFB diagnostic system showed 100% specificity for both the HBV-*S* gene and the HCV 5′-UTR gene, and no cross-reaction was detected. Furthermore, the HBV&HCV-MCDA-AuNPs-LFB assay can detect the target genes (HBV-*S* and HCV-5′-UTR) with as low as 10 copies of gene-containing plasmid template per test. Although the synthetic plasmid (containing target gene) can beused as positive controls for evaluating the LoD of nucleic acid detection technologies. Different templates and sample types may affect the LoD of the same approach (36, 37). The use of quantified standard nucleic acids derived from serum specimens as templates may enable a more comprehensive assessment of our assay’s sensitivity. In addition, the feasibility of our assay was validated using clinical serum samples from suspected HBV- and/or HCV-infected patients. Comparing with the HBV-qPCR method, the sensitivity and specificity of our assay were 100.00% (95% CI: 94.04% to 100.00%) and 100% (95% CI: 92.45% to 100.00%), respectively. Similarly, in comparison with HCV-qPCR methodology, the assay maintained perfect diagnostic performance with 100% sensitivity (95% CI: 91.78% to 100%) and 100% specificity (95% CI: 94.40% to 100%). The data confirmed that our novel assay can effectively identify HBV and HCV agents in clinical practice. To enable a more comprehensive evaluation of our assay, clinical samples representing various HBV/HCV genotypes with low viral copy numbers should be collected and tested. Furthermore, these samples also need to be validated through direct sequencing in subsequent studies.

Isothermal amplification techniques, such as recombinase polymerase amplification (RPA) and loop-mediated isothermal amplification (LAMP), have also been used to detect HBV and HCV (Table 5). Zhang et al. used RPA and detected as few as 10 copies per test for HBV (38). Chia et al. applied an assay based on RPA to detect HCV and identified a LoD of 25 copies per test (39). Vanhomwegen et al. used a method based on LAMP to detect HBV and identified 40 IU/mL–400 IU/mL (40). Hongjaisee et al. applied a LAMP-based method and detected 10 ng–100 ng per test for the HCV agent (41). In addition, we also used the MCDA-based isothermal amplification technique to identify HBV and HCV, respectively, with a LoD as low as 5 IU and 10 copies per test, respectively (27, 28). In the present study, we first used a novel method based on multiplex-MCDA and AuNPs-LFB platform, which can simultaneously identify HBV and HCV in a single test.

**TABLE 5:** Comparison of the commonly used isothermal amplification techniques for HBV/HCV detection

Pathogen	Techniques^*a*^	LoD	Specificity (%)	Detection time (min)	Reference
HBV	RPA-based assay	10 copies per test	100	70	Zhang et al. (38)
HCV	RPA-based assay	25 copies per test	100	17	Chia et al. (39)
HBV	LAMP-based assay	40–400 IU per milliliter	91.5	60	Vanhomwegen et al. (40)
HCV	LAMP-based assay	10–100 ng per test	100	60	Hongjaisee et al. (41)
HBV	MCDA-based assay	5 IU per test	100	Within 80	Chen Xu et al. (28)
HCV	MCDA-based assay	10 copies per test	100	Within 35	Chen Xu et al. (27)
HBV and HCV	MCDA-based assay	10 copies per test	100	Within 50	Current study

^*a*^ RPA, recombinase polymerase amplification; LAMP, loop-mediated isothermal amplification.

For visual and rapid analysis of multiplex-MCDA products, the AuNPs-LFB was devised and constructed in our study. AuNPs-LFB is a paper-based diagnostic platform that has enormous promise for POC testing owing to its high sensitivity, good selectivity, easy construction, visual readout, and cost savings (42, 43), and it has been widely used to test various analytes, such as infectious agents, nucleic acids, and proteins (44). In this study, the AuNPs-LFB can rapidly, visually, and simultaneously monitor multiplex-MCDA products by immobilizing anti-FAM, anti-digoxin, and BSA-biotin on the NC membrane of the biosensor strip, respectively. Here, agarose gel electrophoresis, real-time turbidity, and visual detection reagent (MG) were also used to analyze multiplex-MCDA products. However, the former two methods require specific instruments, and the MG approach was ambiguous when the MCDA product concentrations were low (45). Moreover, all of the aforementioned techniques are inadequate for simultaneously detecting two target genes in a single test. In our study, we first integrated multiplex-MCDA isothermal amplification with a visual AuNPs-LFB diagnostic platform for simultaneously identifying HBV and HCV agents. In addition, AuNPs-LFB is inexpensive (~US$2.0 per test). Hence, the total cost of each test, including nucleic acid extraction (~US$0.5 per test), MCDA (~US$1.5 per test), and AuNPs-LFB interpretation (~US$2.0 per test), was approximately US$4.0 per test.

Our assay also has several limitations. First, our HBV- and HCV-MCDA primers only specifically identified HBV- and HCV-dominant genotypes in China (HBV genotypes B, C, D, B/C recombinant, and C/D recombinant; HCV subtypes 1b, 2a, 3a, 3b, and 6a). Further improvement of the HBV‐MCDA and HCV-MCDA primers is needed because HBV and HCV have high genetic heterogeneity. Second, analyzing the MCDA results using the AuNPs-LFB assay required the opening of the reaction tube, which increased the risk of carryover contamination.

In conclusion, we integrated multiplex-MCDA isothermal amplification with a visual AuNPs-LFB analysis platform to successfully create a novel HBV&HCV-MCDA-AuNPs-LFB assay for simultaneous accurate, sensitive, rapid, inexpensive, and visual identification of HBV and HCV agents. Our HBV&HCV-MCDA-AuNPs-LFB assay had 10 copy LoDs for HBV and HCV and showed no cross-reactivity with other pathogens. The whole diagnostic process can be completed within 50 min with no need for specific devices. Hence, our assay can potentially serve as a useful POC diagnostic tool for the identification of HBV and HCV infection, particularly in low- and middle-income regions.

## MATERIALS AND METHODS

### Clinical sample preparation and standard plasmid construction

A total of 107 serum samples were collected from patients with suspected HBV and/or HCV infections at the Second Affiliated Hospital Guizhou University of Traditional Chinese Medicine from May 2023 to February 2024. The nucleic acids from each sample were extracted using a TIANamp Virus DNA/RNA Fast Kit (TIANGEN BIOTECH Co., Ltd., Beijing, China). In brief, each 200 µL of serum sample was treated with 20 µL of protease K, 250 µL of the lysis agent RLC, and 250 µL of isopropanol and incubated at 56°C for 5 min. Then, the nucleic acids were enriched in a centrifugal RNase-free absorption column. After washing with PWT buffer, the nucleic acids were eluted in 50 µL of RNase-free ddH_2_O. The extracted nucleic acids were stored at −80°C for subsequent analysis.

The full-length HBV-*S* gene sequences of five dominant genotypes in China (B, C, D, recombinant B/C, recombinant C/D) were downloaded from the GenBank database (respective GenBank accession nos. AB014366.1, AB014360.1, AB090268.1, KC774178.1, AY800249.1) (46) (https://www.ncbi.nlm.nih.gov). The full-length HCV-5′-UTR gene sequences of five dominant subtypes in China (1b, 2a, 3a, 3b, and 6a) were downloaded from the GenBank database (respective GenBank accession nos. EU781827.1, HQ639944.1, D17763.1, JQ065709.1, and AY859526.1) (47). Each sequence was synthetically produced, cloned, and inserted into the customized pUC57 plasmid. The constructed plasmids were used as positive controls. The concentration of each plasmid was 10^8^ copies/mL.

### MCDA primer design and synthesis

Based on the principle of the MCDA reaction, HBV- and HCV-MCDA primers were designed to target the HBV-*S* and HCV-5′-UTR genes, respectively. The *S* genes from HBV genotypes B, C, D, B/C recombinant, and C/D recombinant and the 5′-UTR genes from HCV subtypes 1b, 2a, 3a, 3b, and 6a were aligned using DNASTAR software (Madison, USA) separately. The conserved sequences were used for MCDA primer design. Each set of MCDA primers consisted of two displacement primers (F1 and F2), two cross primers (CP1 and CP2), and six amplification primers (C1, C2, D1, D2, R1, and R2). The specificity of the HBV- and HCV-MCDA primer sets was validated using the National Center for Biotechnology Information basic local alignment search tool. The details of the MCDA primer locations, sequences, and modifications are shown in Fig. 2 and Table 1. All oligomers were synthesized and purified at high-performance liquid chromatography grade by TsingKe Biotech. Co., Ltd. (Beijing, China).

### Nanoparticle-based biosensor preparation

The AuNPs-LFB, illustrated in Fig. 1A, was constructed as described in previous reports (48). Briefly, the AuNPs-LFB comprised four sections, including a sample pad, a conjugate pad, an NC membrane, and an absorbent pad, all of which were laminated and packaged on a plastic adhesive backing card. Crimson dye-coated SA-AuNPs were assembled on the conjugate pad. Anti-FAM, anti-digoxin, and biotinylated bovine serum albumin (biotin-BSA) were sprayed onto the NC membrane for TL1, TL2, and the CL, respectively, and each line was separated by 5 mm. The AuNPs-LFB developed in the current study can simultaneously detect two targets. The AuNPs-LFB used in this study were manufactured by Tian-Jin HuiDeXin Biotech Co., Ltd. (Tianjin, China) according to our design (Fig. 1A) and stored at room temperature until use.

### MCDA assay

Standard MCDA reactions were conducted to evaluate the feasibility of the two sets of MCDA primers. The one-step single MCDA reaction for HBV or HCV was performed in a 25 µL volume. Briefly, 12.5 µL of 2 × reaction buffer (2 M betaine, 16 mM MgSO_4_, 40 mM KCl, 20 mM (NH_4_)_2_SO_4_, 40 mM Tris-HCl [pH 8.8], and 0.2% Tween-20), 3 µL of dNTP mixture (10 mM), 1 µL of *Bst* 2.0 DNA polymerase (8 U), 1 µL of avian myeloblastosis virus (AMV) reverse transcriptase (10 U) (only for RNA templates), 0.4 µM each of F1 and F2, 1.6 µM each of CP1 and CP2, 0.8 µM each of C1 or C1* (for AuNPs-LFB only), C2, D1, or D1* (for AuNPs-LFB only), D2, R1, and R2, 2 µL of colorimetric indicator (MG) (for colorimetry only), 1 µL of standard plasmid template (5 µL of clinical sample template), and double-distilled water (ddH_2_O) were added to yield a total volume of 25 µL. The reactions were performed at 65°C for 60 min.

The one-step multiplex-MCDA reaction was also performed in a 25 µL mixture. The reaction mixture consisted of 12.5 µL 2 × reaction buffer (2 M betaine, 16 mM MgSO_4_, 40 mM KCl, 20 mM (NH_4_)_2_SO_4_, 40 mM Tris-HCl [pH 8.8], and 0.2% Tween-20), 3 µL dNTP mixture (10 mM), 2 µL *Bst* 2.0 DNA polymerase (8 U), 1 µL AMV reverse transcriptase (10 U) (only for RNA templates), 0.2 µM each *S*-F1 and *S*-F2, 0.8 µM each *S*-CP1 and *S*-CP2, 0.4 µM each *S*-C1 or *S*-C1* (for AuNPs-LFB only), *S*-C2, *S*-D1, or *S*-D1* (for AuNPs-LFB only), *S*-D2, *S*-R1, and *S*-R2, 0.4 µM each 5′-UTR-F1 and 5′-UTR-F2, 1.6 µM each 5′-UTR-CP1 and 5′-UTR-CP2, 0.8 µM each 5′-UTR-C1 or 5′-UTR-C1* (for AuNPs-LFB only), 5′-UTR-C2, 5′-UTR-D1, or 5′-UTR-D1* (for AuNPs-LFB only), 5′-UTR-D2, 5′-UTR-R1, and 5′-UTR-R2, 2 µL colorimetric indicator (MG) (for colorimetry only), 1 µL of each standard plasmid template (5 µL of the clinical sample template), and then add double-distilled water (ddH_2_O) up to 25 µL. The reactions were performed at 65°C for 60 min.

Several monitoring approaches, including agarose gel electrophoresis, visual detection reagent (MG) (0.25 µM), real-time turbidity (LA-500, Eiken Chemical Co., Ltd., Japan), and AuNPs-LFB, were used to analyze the MCDA reaction products. For agarose gel electrophoresis detection, the agarose gel presented ladder-like bands indicating a positive outcome, whereas the lack of bands indicated a negative result. For visual MG analysis, changing the reaction mixture to light green suggested a positive outcome, whereas colorless mixtures indicated a negative result. For turbidity measurements, a real-time turbidity value >0.1 indicated a positive outcome. For AuNPs-LFB identification, an aliquot of 1 µL of each MCDA reaction mixture and 100 µL of running buffer (100 mM PBS, 1% Tween 20, pH 7.4) were added simultaneously to the sample pad. Then, the samples flowed along the biosensor via capillary action. Finally, the presence or absence of the targets could be tested at the NC membrane (crimson red line) within 2 min. Both the CL and TL1 simultaneously appeared on the AuNPs-LFB strip, demonstrating an HBV-positive result. Both the CL and TL2 simultaneously turned red on the biosensor, indicating an HCV-positive outcome. For a negative outcome, only CL was present on the AuNPs-LFB strips.

### Optimization of the reaction temperature and time for the HBV&HCV-MCDA-AuNPs-LFB assay

The reaction temperature is important for improving the efficiency of MCDA. In this study, the optimal reaction temperature for our assay was tested by incubating HBV-MCDA and HCV-MCDA at 63 to 70°C at 1°C intervals under the standard MCDA reaction. HBV-genotype-B and HCV-subtype-1b standard plasmids (1 × 10^4^ copies per test) were used as templates, respectively. The reaction results were monitored by using real-time turbidity. In addition, the optimal reaction time for multiplex MCDA was determined to range from 25 to 40 min with 5 min intervals under the optimal reaction temperature. The MCDA amplicons were analyzed using the AuNPs-LFB assay. Each test was performed independently in triplicate.

### Sensitivity of the HBV&HCV-MCDA-AuNPs-LFB assay

The plasmid templates, including HBV-genotype-B and HCV-subtype-1b plasmids, were serially diluted 10-fold, ranging from 2.0 × 10^4^ copies to one copy per test, to determine the LoD of the HBV&HCV-MCDA assay. The assays were performed under optimal reaction conditions, and the amplicons were analyzed with AuNPs-LFBs using distilled water as the BC. Each test was performed at least thrice.

### Specificity of the HBV&HCV-MCDA-AuNPs-LFB assay

The specificity and selectivity of the multiplex-MCDA-AuNPs-LFB assay were verified by using five HBV-S plasmids (genotypes B, C, D, recombinant B/C, recombinant C/D), five HCV-5′-UTR plasmid templates (HCV subtypes 1b, 2a, 3b, 6a, and 3a), HBV/HCV-positive clinical samples (confirmed by real-time PCR), and other pathogens, including herpes simplex virus, parainfluenza virus, HAV, coxsackievirus CAV16, human enterovirus EV71, influenza A virus, influenza B virus, human papilloma virus, HIV, *Mycobacterium leprae*, *Mycobacterium tuberculosis*, *Haemophilus influenzae*, *Cryptococcus neoformans,* and their mixtures (Table 2). All the microbial nucleic acid templates used in this study were isolated using Bacterial Genomic DNA Extraction Kits or Virus DNA/RNA Extraction Kits (Xi'an Tianlong Technology Co., Ltd., Xi'an, China), and the concentrations were determined with a NanoDrop ND-2000 (Beijing, China) at A260/280. The concentrations of each nucleic acid template more than 10^4^ copies/mL. The assays were performed under optimal reaction conditions with the same sets of multiplex-MCDA degenerate primers. All of the amplicons were detected using the AuNPs-LFB assay, with distilled water serving as the BC. Each test was performed independently in triplicate on different days.

### Feasibility of the HBV&HCV-MCDA-AuNPs-LFB assay in clinical specimens

To confirm the feasibility of the multiplex-MCDA-AuNPs-LFB assay in clinical practice, 107 serum samples from patients with suspected HBV and/or HCV infection were used. The nucleic acids were rapidly isolated using a TIANamp Virus DNA/RNA Fast Kit (TIANGEN BIOTECH Co., Ltd., Beijing, China) according to the manufacturer′s instructions. Then, these templates were tested simultaneously using commercially available real-time TaqMan PCR Kits (Xi′an Tianlong Technology Co., Ltd., Xi′an, China) and our HBV&HCV-MCDA-AuNPs-LFB assay. The experiments were independently conducted in triplicate. HBV >5 IU (~30 copies) and HCV >50 IU (~45 copies) were regarded as positive outcomes according to the manufacturer′s recommendations. The outcomes of our HBV&HCV-MCDA-AuNPs-LFB assay were compared with those of a real-time PCR approach. The predictive sensitivity and specificity values were calculated by using the online tool from MedCalc (http://www.medcalc.org/calc/diagnostic_test.php) (49).

## Data Availability

The original contributions presented in the study are included in the article/supplementary material, further inquiries can be directed to the corresponding author.

## SUPPLEMENTAL MATERIAL

Supplemental material spectrum.01738-24-s0001.docx Table S1, Fig. S1 and S2.

ASM does not own the copyrights to Supplemental Material that may be linked to, or accessed through, an article. The authors have granted ASM a non-exclusive, world-wide license to publish the Supplemental Material files. Please contact the corresponding author directly for reuse.
