# Supplementary material for: Nanoparticle-based biosensor integrated with multiple cross-displacement amplification for visual and rapid identification of hepatitis B virus and hepatitis C virus
Source: Microbiol Spectr. 2025 Apr 15;13(5):e01738-24. doi: 10.1128/spectrum.01738-24 (PMC12054127; doi:10.1128/spectrum.01738-24)
Supplement: Supplemental material — Table S1, Fig. S1 and S2. [file spectrum.01738-24-s0001.docx]

**Supplementary Material**

**Nanoparticles-based biosensor integrated with multiple cross-displacement amplification for visual and rapid identification of hepatitis B virus and hepatitis C virus**

Hang Zhang^a,bΔ^, Yuanfang Shi^bΔ^, Zengguang Wu^b,c^, Qi Zhao^d^, Yu Wang^e^, Xinggui Yang^f^, Yan Tan^g^, Yi Wang^h*^, Zhenghua Xiao^b,d*^, Xu Chen^b,c*^

^a^Clinical Laboratory, the Second Affiliated Hospital, Guizhou University of Traditional Chinese Medicine, Guiyang, Guizhou, 550003, People′s Republic of China

^b^The Second Clinical Medical College, Guizhou University of Traditional Chinese Medicine, Guiyang, Guizhou, 550003, People′s Republic of China

^c^Department of Scientific Research, the Second Affiliated Hospital, Guizhou University of Traditional Chinese Medicine, Guiyang, Guizhou, 550003, People′s Republic of China

^d^Department of gastroenterology, the Second Affiliated Hospital, Guizhou University of Traditional Chinese Medicine, Guiyang, Guizhou, 550003, People′s Republic of China

^e^Department of Clinical Laboratory Centre, The First People′s Hospital of Guiyang, Guiyang, Guizhou, 55004, People′s Republic of China

^f^Experimental Center, Guizhou Provincial Centre for Disease Control and Prevention, Guiyang, Guizhou, 550004, People′s Republic of China

^g^Clinical Laboratory, Guizhou Provincial Center for Clinical Laboratory, Guiyang, Guizhou 550002, People′s Republic of China

^h^Experimental Research Center, Capital Institute of Pediatrics, Beijng 100020, People’s Republic of China

^Δ^Drs. Hang Zhang and Yuanfang Shi have contributed equally to this work.

^*^Corresponding author:

Xu Chen, E-mail: [xuchen1220@126.com](mailto:xuchen1220@126.com) (Handing the correspondence)

Zhenghua Xiao, E-mail: xiaozhenghua097@126.com

Yi Wang, E-mail: [wildwolf0101@163.com](mailto:wildwolf0101@163.com)

**Table S1** Comparison of HBV&HCV-MCDA-AuNPs-LFB and qPCR methods for assessment of HBV and HCV in clinical samples

| **Sample No.** | **qPCR^a^ results (copies/ml)** | | **HBV&HCV-MCDA-AuNPs-LFB results** | |
| --- | --- | --- | --- | --- |
|  | **HBV** | **HCV** | **HBV** | **HCV** |
| Test 1 | 2.15×10^4^ | **—** | + | **—** |
| Test 2 | 1.78×10^6^ | **—** | + | **—** |
| Test 3 | 3.61×10^3^ | **—** | + | **—** |
| Test 4 | 1.82×10^4^ | **—** | + | **—** |
| Test 5 | 7.81×10^6^ | **—** | + | **—** |
| Test 6 | 2.91×10^3^ | **—** | + | **—** |
| Test 7 | 8.12×10^4^ | **—** | + | **—** |
| Test 8 | 1.61×10^3^ | **—** | + | **—** |
| Test 9 | 9.23×10^3^ | **—** | + | **—** |
| Test 10 | 8.12×10^4^ | **—** | + | **—** |
| Test 11 | 7.13×10^2^ | **—** | + | **—** |
| Test 12 | 1.26×10^5^ | **—** | + | **—** |
| Test 13 | 4.14×10^3^ | **—** | + | **—** |
| Test 14 | 5.31×10^6^ | **—** | + | **—** |
| Test 15 | 4.13×10^3^ | **—** | + | **—** |
| Test 16 | 4.41×10^6^ | **—** | + | **—** |
| Test 17 | 9.43×10^2^ | **—** | + | **—** |
| Test 18 | 1.13×10^5^ | **—** | + | **—** |
| Test 19 | 7.80×10^2^ | **—** | + | **—** |
| Test 20 | 5.34×10^4^ | **—** | + | **—** |
| Test 21 | 3.62×10^5^ | **—** | + | **—** |
| Test 22 | 1.83×10^4^ | **—** | + | **—** |
| Test 23 | 2.45×10^7^ | **—** | + | **—** |
| Test 24 | 3.18×10^3^ | **—** | + | **—** |
| Test 25 | 5.63×10^4^ | **—** | + | **—** |
| Test 26 | 9.22×10^2^ | **—** | + | **—** |
| Test 27 | 5.38×10^3^ | **—** | + | **—** |
| Test 28 | 7.27×10^6^ | **—** | + | **—** |
| Test 29 | 2.89×10^5^ | **—** | + | **—** |
| Test 30 | 7.31×10^4^ | **—** | + | **—** |
| Test 31 | 9.16×10^5^ | **—** | + | **—** |
| Test 32 | 5.31×10^4^ | **—** | + | **—** |
| Test 33 | 1.95×10^6^ | **—** | + | **—** |
| Test 34 | 3.89×10^3^ | **—** | + | **—** |
| Test 35 | 7.32×10^5^ | **—** | + | **—** |
| Test 36 | 3.67×10^4^ | **—** | + | **—** |
| Test 37 | 9.81×10^2^ | **—** | + | **—** |
| Test 38 | 6.38×10^4^ | **—** | + | **—** |
| Test 39 | 3.91×10^5^ | **—** | + | **—** |
| Test 40 | 8.42×10^2^ | **—** | + | **—** |
| Test 41 | 4.82×10^4^ | **—** | + | **—** |
| Test 42 | 9.17×10^3^ | **—** | + | **—** |
| Test 43 | **—** | 3.03×10^6^ | **—** | + |
| Test 44 | **—** | 4.51×10^4^ | **—** | + |
| Test 45 | **—** | 7.21×10^3^ | **—** | + |
| Test 46 | **—** | 7.19×10^5^ | **—** | + |
| Test 47 | **—** | 6.24×10^4^ | **—** | + |
| Test 48 | **—** | 5.31×10^3^ | **—** | + |
| Test 49 | **—** | 2.18×10^6^ | **—** | + |
| Test 50 | **—** | 4.25×10^4^ | **—** | + |
| Test 51 | **—** | 5.21×10^3^ | **—** | + |
| Test 52 | **—** | 2.41×10^7^ | **—** | + |
| Test 53 | **—** | 6.18×10^4^ | **—** | + |
| Test 54 | **—** | 8.26×10^4^ | **—** | + |
| Test 55 | **—** | 5.32×10^5^ | **—** | + |
| Test 56 | **—** | 8.69×10^3^ | **—** | + |
| Test 57 | **—** | 5.18×10^4^ | **—** | + |
| Test 58 | **—** | 7.92×10^4^ | **—** | + |
| Test 59 | **—** | 6.36×10^5^ | **—** | + |
| Test 60 | **—** | 4.21×10^3^ | **—** | + |
| Test 61 | **—** | 6.60×10^2^ | **—** | + |
| Test 62 | **—** | 1.07×10^4^ | **—** | + |
| Test 63 | **—** | 3.91×10^6^ | **—** | + |
| Test 64 | **—** | 2.09×10^5^ | **—** | + |
| Test 65 | **—** | 4.32×10^7^ | **—** | + |
| Test 66 | **—** | 6.91×10^5^ | **—** | + |
| Test 67 | **—** | 6.21×10^6^ | **—** | + |
| Test 68 | **—** | 2.09×10^4^ | **—** | + |
| Test 69 | **—** | 3.75×10^6^ | **—** | + |
| Test 70 | 7.40×10^3^ | 3.87×10^6^ | + | + |
| Test 71 | 5.94×10^7^ | 1.95×10^3^ | + | + |
| Test 72 | 7.01×10^3^ | 4.82×10^4^ | + | + |
| Test 73 | 6.82×10^2^ | 8.01×10^3^ | + | + |
| Test 74 | 3.71×10^5^ | 6.69×10^3^ | + | + |
| Test 75 | 1.05×10^3^ | 7.24×10^5^ | + | + |
| Test 76 | 7.58×10^6^ | 4.06×10^3^ | + | + |
| Test 77 | 4.82×10^6^ | 6.72×10^4^ | + | + |
| Test 78 | 6.92×10^5^ | 8.90×10^3^ | + | + |
| Test 79 | 2.63×10^3^ | 5.90×10^5^ | + | + |
| Test 80 | 7.83×10^2^ | 2.47×10^5^ | + | + |
| Test 81 | 4.18×10^6^ | 6.82×10^3^ | + | + |
| Test 82 | 4.80×10^1^ | **—** | + | **—** |
| Test 83 | 5.70×10^1^ | **—** | + | **—** |
| Test 84 | 3.60×10^1^ | **—** | + | **—** |
| Test 85 | 4.20×10^1^ | **—** | + | **—** |
| Test 86 | 3.80×10^1^ | **—** | + | **—** |
| Test 87 | 3.20×10^1^ | **—** | + | **—** |
| Test 88 | **—** | 5.60×10^1^ | **—** | + |
| Test 89 | **—** | 5.10×10^1^ | **—** | + |
| Test 90 | **—** | 6.80×10^1^ | **—** | + |
| Test 91 | **—** | 4.80×10^1^ | **—** | + |
| Test 92-107 | **—** | **—** | **—** | **—** |

Notice: ^a^qPCR, the qPCR diagnosis was performed using commercially available real-time TaqMan PCR kits for HBV and HCV (Xi′an Tianlong Technology Co. Ltd., Xi′an, China). The concentrations of HBV > 5 IU (~30 copies/ml) and HCV > 50 IU (~45 copies/ml) were regarded as positive based on the manufacturer’s recommendations.

+, Positive; —, Negative.

**Figure legends**

**
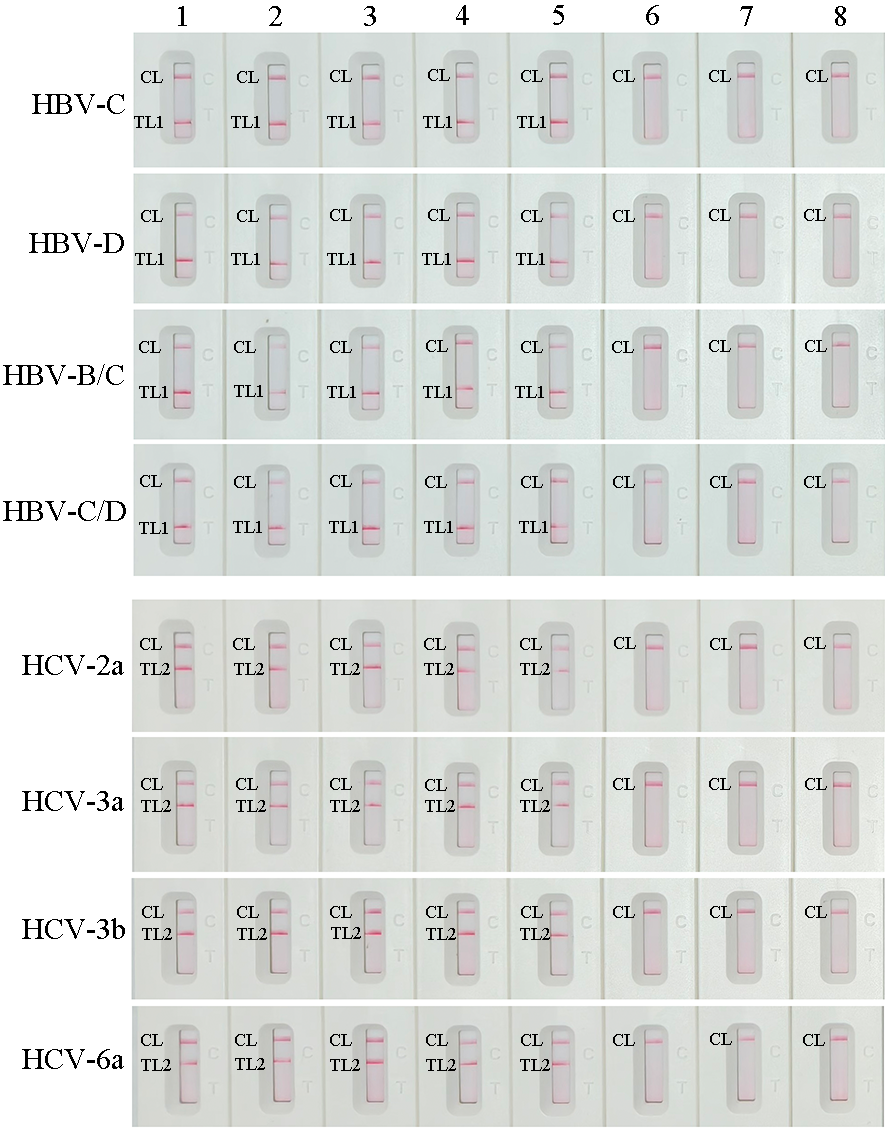
**

**Fig. S1 Sensitivity analysis of HBV&HCV-MCDA-AuNPs-LFB assay with different HBV/HCV genotype nucleic acid templates**

Other four HBV genotype (C, D, recombinant B/C, recombinant C/D) and four HCV subtype (2a, 3a, 3b, and 6a) plasmid templates were tested under the optimal reaction conditions (64°C, 35 min). Biosensor 1-8 represented the plasmid amounts of 2.0×10^4^ copies, 2.0×10^3^ copies, 2.0×10^2^ copies, 20 copies, 10 copies, 5 copies, 1 copy per reaction, and distilled water (DW), respectively. The results were shown that the LoD of each HBV/HCV genotype was 10 copies/test. CL: control line, TL1: test line one, TL2: test line two.


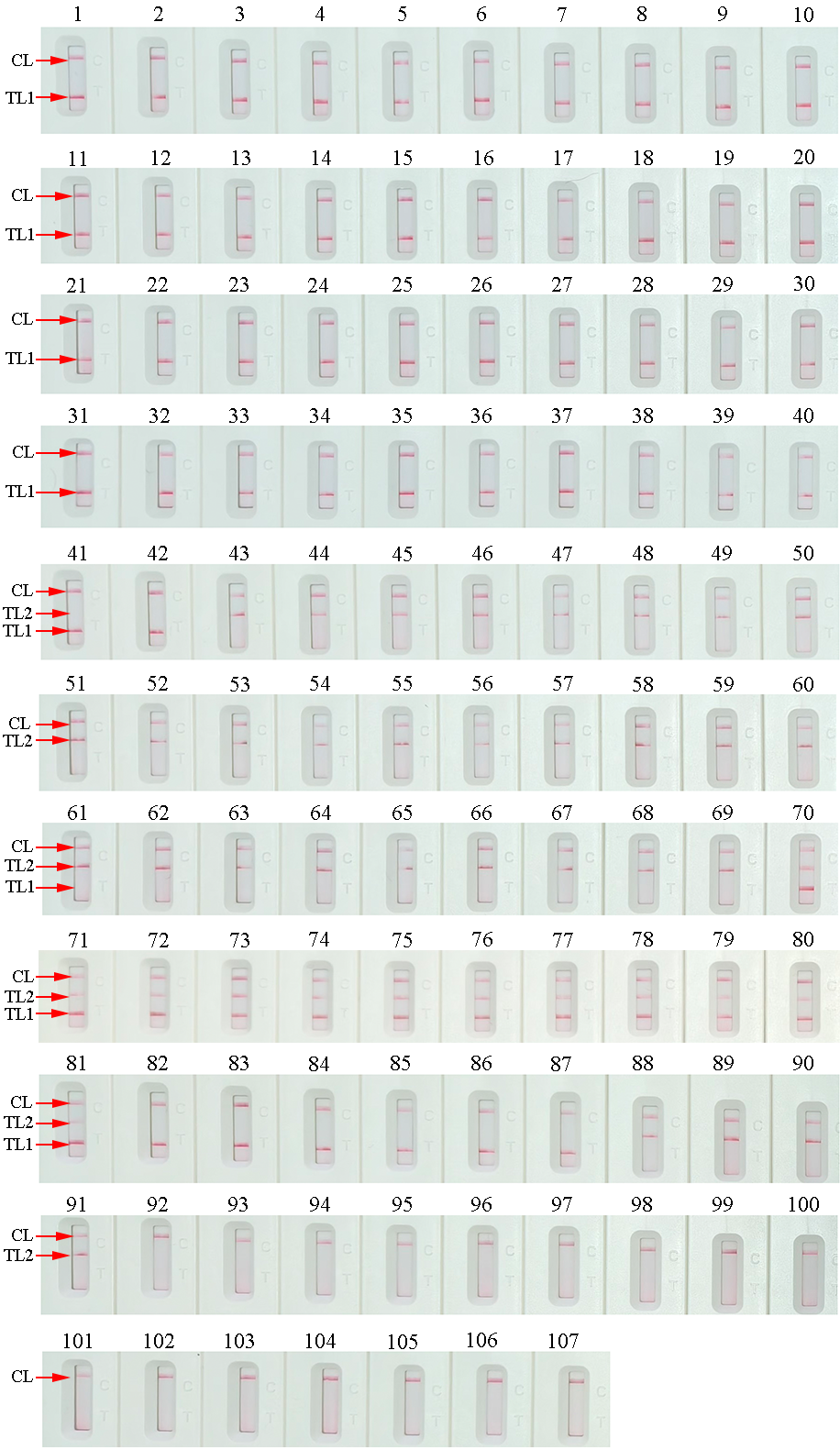


**Fig. S2 The results of HBV&HCV-MCDA-AuNPs-LFB assay in clinical specimens**

107 serum samples with suspected HBV and/or HCV infection were tested using HBV&HCV-MCDA-AuNPs-LFB assay. Both the CL and TL1 simultaneously appeared on the AuNPs-LFB strip, demonstrating an HBV-positive result. Both the CL and TL2 simultaneously turned red on the biosensor, indicating an HCV-positive outcome. For a negative outcome, only CL was present on the AuNPs-LFB strips. CL: control line, TL1: test line one, TL2: test line two.
